# Coherence based graph convolution network for motor imagery-induced EEG after spinal cord injury

**DOI:** 10.3389/fnins.2022.1097660

**Published:** 2023-01-13

**Authors:** Han Li, Ming Liu, Xin Yu, JianQun Zhu, Chongfeng Wang, Xinyi Chen, Chao Feng, Jiancai Leng, Yang Zhang, Fangzhou Xu

**Affiliations:** ^1^International School for Optoelectronic Engineering, Qilu University of Technology, Shandong Academy of Sciences, Jinan, China; ^2^Rehabilitation Center, Qilu Hospital of Shandong University, Jinan, China

**Keywords:** electroencephalogram, motor imagery, brain-computer interface, coherence-based graph convolutional network, spinal cord injury

## Abstract

**Background:**

Spinal cord injury (SCI) may lead to impaired motor function, autonomic nervous system dysfunction, and other dysfunctions. Brain-computer Interface (BCI) system based on motor imagery (MI) can provide more scientific and effective treatment solutions for SCI patients.

**Methods:**

According to the interaction between brain regions, a coherence-based graph convolutional network (C-GCN) method is proposed to extract the temporal-frequency-spatial features and functional connectivity information of EEG signals. The proposed algorithm constructs multi-channel EEG features based on coherence networks as graphical signals and then classifies MI tasks. Different from the traditional graphical convolutional neural network (GCN), the C-GCN method uses the coherence network of EEG signals to determine MI-related functional connections, which are used to represent the intrinsic connections between EEG channels in different rhythms and different MI tasks. EEG data of SCI patients and healthy subjects have been analyzed, where healthy subjects served as the control group.

**Results:**

The experimental results show that the C-GCN method can achieve the best classification performance with certain reliability and stability, the highest classification accuracy is 96.85%.

**Conclusion:**

The proposed framework can provide an effective theoretical basis for the rehabilitation treatment of SCI patients.

## 1. Introduction

Spinal cord injury (SCI) is a catastrophic disease, which can lead to the loss of motor and sensory functions. In severe cases, it can lead to the interruption of some routes connecting the brain and limbs. Many SCI patients experience chronic pain that is difficult to treat (Jensen et al., 2005; Cardenas and Jensen, 2006). There are more than 3.7 million SCI patients in China, with an annual incidence of about 90,000 new patients per year, and the annual incidence rate is 17.9 to 60.2 people/million people. The motor dysfunction caused by SCI not only brings serious physical and psychological harm to the patients themselves but also imposes a huge economic burden on society and families (Aguilar et al., 2010). To reduce the harm caused by SCI patients, researchers have explored the changes in the brain of SCI patients after chronic injury. Studies have shown that chronic pain associated with SCI may be related to changes in brain activity reflected in the electroencephalogram (EEG) (López-Larraz et al., 2012), the differences in EEG reflect some extent the experience of pain. At present, the EEG studies after SCI have the following aspects, the changes in event-related synchronization/desynchronization (ERD/ERS) after SCI, the changes in power spectrum occurring after SCI, the changes in network characteristics of brain networks after SCI, the changes in performance of brain-computer interface (BCI) systems after SCI.

**FIGURE 1:**
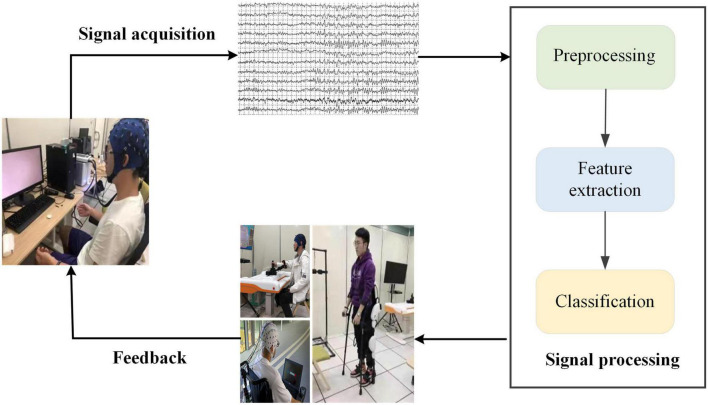
Block diagram of motor imagery (MI)-based brain-computer interface (BCI) system. The system consists of four main parts: signal acquisition, signal processing, application, and feedback.

In recent years, motor imagery (MI)-based BCI systems have become the focus of attention in the field of rehabilitation medicine, such as neuro-robotics and neuro-prosthetic device control (Iturrate et al., 2009; Millán et al., 2010; Escolano et al., 2011). The MI-BCI system aims to deliver MI task interventions for SCI patients and to assist in the formulation of rehabilitation programs to alleviate patient suffering. As shown in Figure 1, MI-based BCI systems mainly consist of four parts, signal acquisition, signal processing, application, and feedback, use brain signals to control external assistive devices (Collinger et al., 2014). Traditional SCI rehabilitation training, which lacks the active participation of the patient and the reconstruction of neural pathways is slow, is mainly based on the passive movement by a patient to achieve the recovery of muscle strength and the reconstruction of neural pathways. BCI technology for SCI rehabilitation takes into account the functional coupling between the patient’s MI intentions and the actual motor effects. It is more in line with the theoretical requirements of neurological reconstruction and can promote faster and better motor recovery in SCI patients. In the study of MI-based BCI systems for SCI patients, Müller-Putz et al. (2014) achieved an average accuracy of 66.1% using common spatial pattern (CSP) algorithms and linear discriminant analysis (LDA) for classification. Xu et al. (2022b) proposed Modified Graph Convolutional Neural Network (M-GCN) method, which performs time-frequency processing of data by modified S-transform (MST) to improve decoding performance.

MI refers to the procedure of imagining limb actions without actual limb movements (Zhang et al., 2018). Related studies have shown that the sensorimotor cortical areas of the brain stimulated by MI are the same as those stimulated by actual limb movement (Azab et al., 2019). MI is considered to be a process involving multiple higher cognitive functions (Rimbert et al., 2019). The brain is a complex network, the information related to MI is both spatially independent and interconnected, and brain network correlation methods can investigate the functional mechanisms of MI. EEG coherence provides an important estimate of the functional interactions between neural systems in each frequency band and is often used to assess the functional connectivity of the human cortex (Srinivasan et al., 2007). Due to its targeting of functional mechanisms, more and more people have begun to pay attention to coherence networks and have used them to decode relevant cognitive functions. Benefiting from MI therapy, patients with cortical damage have better performance in functional recovery, and BCI investigators achieve higher classification accuracy (Weiskopf et al., 2004).

Hinton and Salakhutdinov (2006) published a paper in Science on the dimensionality reduction of data with neural networks, which attracted great attention. AlexNet performed brilliantly in the ImageNet image recognition competition (Krizhevsky et al., 2017), which started the boom of deep learning (DL). Relying on advances in various aspects such as large data volume, non-convex optimization, hardware computation, and network structure, DL methods represented by convolutional neural networks (CNN), recurrent neural networks (RNN), and generative adversarial networks (GAN) achieved excellent results in the processing of regular data such as images, audio, video, and text. Deep neural networks have achieved great success in data processing, more and more people have begun to apply them to BCI systems. In 2022, Roy (2022) proposed a multi-scale CNN (MS-CNN) model with intrinsic feature integration for motor image EEG subject classification in the BCI system. Supakar et al. (2022) used the recurrent neural networks-Long Short-Term Memory (CNN-LSTM) method to analyze EEG signal data to diagnose schizophrenia. Xu et al. (2022a) applied the deep convolution generative adversarial network (DCGAN) to rehabilitation-based BCI systems.

Deep learning frameworks generally have large models and many parameters. They need higher amounts of training and requirements for computing conditions. How to extend DL methods to irregular data structures is a current research hotspot in the field of neural networks. Data processing based on graph structures mainly involves the representation learning of graph nodes, classification of graph nodes, prediction of edges in graphs, classification of graphs, and so on. Irregular data represented by graphs, such as traffic flow networks with cities as nodes, molecular structure networks with various types of atoms as nodes, and EEG structure networks with electrodes as nodes, are playing an increasingly important role in the storage of data and the description of relationships between entities. To efficiently extract space features on this data structure, graph convolutional network (GCN) is proposed. Chen et al. (2020) proposed the E-GCN method to deeply mine the relationship between EEG channels and to use it for the detection of epileptic EEG signals. Zeng et al. (2020) proposed the hierarchical graph convolution (HGCN) network for classification tasks using topological relationships between each electrode, where power spectral density and continuous wavelet transform features from the raw EEG signal are used as frequency domain inputs.

The above methods only consider EEG channel location relationships and do not explore functional linkages. Considering the working mechanism for the division of labor and cooperation between brain regions, the spatial location relationships and functional linkages of EEG channels do not maintain their consistency (Wang et al., 2019). In this paper, the coherence network-based graph convolution (C-GCN) method is proposed to analyze MI-based EEG data, the main contributions are as follows,

(1) Due to the fact that traditional GCN can only analyze the spatial relationship of channels but not describe the connection of brain functions, the C-GCN algorithm is proposed to represent the temporal-frequency-spatial domain representation of EEG data.
(2) Compared with Support Vector Machine (SVM), CNN, EEGNet, RNN, LSTM, traditional GCN, M-GCN, Graph Attention Network (GAT), and ResGCN, the proposed C-GCN algorithm can obtain the best performance of 96.85% for two-class MI recognition.
(3) The coherence network of EEG data at different frequency bands from SCI patients and healthy subjects is used to perform functional analysis and to provide rehabilitation training guidance for SCI patients.

The rest content is arranged as follows, section “2. Experimental data and experimental paradigm” introduces the experimental data. Section “3. Materials and methods” introduces the pre-processing work and the C-GCN model. Section “4. Results and discussions” shows the experimental results and discussions. Finally, section “4. Conclusion” summarizes the whole paper.

## 2. Experimental data and experimental paradigm

The EEG data used in the experiment were collected from the Department of Physical Medicine and Rehabilitation, Qilu Hospital, Shandong University, and the protocol of this experiment was approved by the Medical Ethics Committee of Qilu Medical College, Shandong University [No. KYLL-2020(KS)-475]. Before the experiment, all subjects signed an informed consent form and were free from habitual medication, alcohol consumption, and cognitive impairment. Experiments were carried out in a closed environment where subjects were undisturbed and attentive, E-Prime software was used for MI stimulation, and 64-lead EEG signal acquisition system was used to capture the subject’s EEG signals. Twenty-five subjects were recruited for the experiment, including 18 SCI patients and 7 healthy subjects, the healthy subjects serving as controls in the experiment.

**FIGURE 2:**
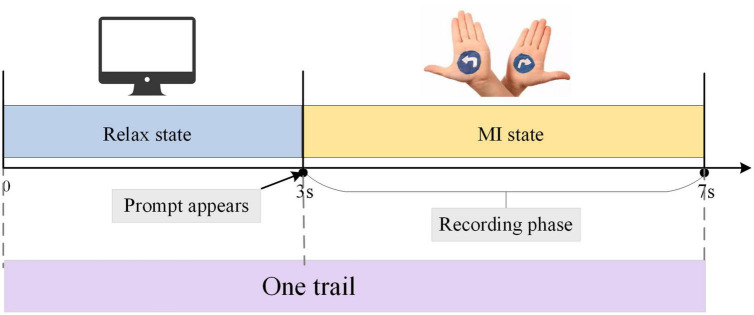
The procedure for one electroencephalogram (EEG) signal acquisition trial. The duration of one trial is 7 s, including 3 s of rest state and 4 s of MI state.

During the MI experiment, the subjects sat in front of the instruction screen. Before each imaginary movement, the screen was blank, which was a rest period to prevent visually evoked potentials, that is, the time interval between two imaginary movements. After the left and right arrow prompts appeared, the MI tasks began. During this time, subjects began to imagine themselves performing left-hand or right-hand movements, and the duration of the imagined movements was 4 s. The experimental paradigm is shown in Figure 2. Each group of experiments consisted of 20 randomly occurring MI tasks, and each subject performed four groups of experiments with a 90 s rest period between every two groups. Each subject performed 80 trials, 40 each of the left-hand MI tasks and the right-hand MI tasks, and the emergence of the left-hand and right-hand MI tasks was randomized.

## 3. Materials and methods

### 3.1. Pre-processing

Electroencephalogram data are pre-processed before being fed into the C-GCN model. Zero-reference processing is performed by the reference electrode normalization technique (REST) (Xu et al., 2014) to obtain artifact-free data. EEG signals are filtered by a fifth-order Butterworth filter with 8–30 Hz to remove noise. Channel selection and data segmentation techniques are also involved in pre-processing procedure.

**FIGURE 3:**
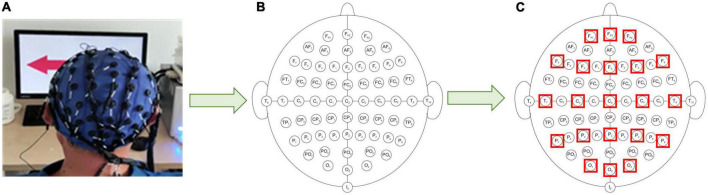
Schematic diagram of electrode position distribution. **(A)** Three-dimensional electrode position distribution. **(B)** Two-dimensional electrode position distribution. **(C)** Experimentally selected electrode position distribution.

MI-related information is generally concentrated in specific frequency bands, therefore during the EEG data filtering process, the EEG data are divided into multiple data bands (μ rhythm, β rhythm, μ, and β rhythms). To reduce the effect of volume conduction between network nodes, 21 electrodes of 64 electrodes (“Fp1”, “Fp2”, “F7”, “F3”, “Fz”, “F4”, “F8”, “T7”, “Cz”, “C4”, “T8”, “P7”, “P3”, “Pz”, “P4”, “P8”, “O1”, “O2”, and “Oz”) are selected for subsequent processing (Li et al., 2016), and the electrode positions select for the experiment are shown in Figure 3. Resting-state EEG shows spontaneous brain activity in the idle state, whereas MI-state EEG records the event-related activity during the desired task. One trial consists of 3 s resting state and 4 s MI state. The MI state of EEG is employed for the analysis of coherence network.

### 3.2. Coherence

The pre-processed EEG data are employed for coherence network construction. Coherence is the squared correlation coefficient (Zhang et al., 2015), which characterizes the connectivity between the brain network channels of the MI tasks. Coherence, which is the degree of linear correlation between two EEG signals *x*(*t*) and *y*(*t*) at specific frequencies, is used to measure the strength of the interaction between each pair of electrodes (Weiss and Mueller, 2003; Murias et al., 2007). High coherence between the two EEG electrodes indicates the contribution of synchronized neuronal oscillations to each electrode, indicating functional integration between neural populations. Low coherence indicates functional separation. The coherence coefficients of the EEG signals *x*(*t*) and *y*(*t*) are defined as,


(1) $$C_{x\,y}=\frac{{\left|P_{x\,y}\,\left(f\right)\right|}^{2}}{P_{x\,x}\,\left(f\right)\,P_{y\,y}\,\left(f\right)}$$

where *P*_*xy*_(*f*) is the cross-spectral density of *x*(*t*) and *y*(*t*), *P*_*xx*_(*f*) and *P*_*yy*_(*f*) are the self-spectral densities of *x*(*t*) and *y*(*t*), respectively. *C*_*xy*_(*f*) is the coherence at frequency *f*.

After the coherence calculation, the coherence is averaged over the corresponding frequency band to obtain the final strength of the connection between the two nodes. The coherence coefficient takes values in the range of 0–1. When the coherence coefficient is closer to 1, the two signals are more coherent. The coherence network has 21 nodes due to the selected 21 channels of the subject. Therefore, the EEG coherence network is constructed by a 21 × 21 weighting matrix.

### 3.3. C-GCN

In traditional convolutional networks, convolution essentially uses a filter with shared parameters to extract spatial features by computing a weighted sum of the central pixel and neighboring pixels. Convolution is an operation between signals on a regular grid. With the production of discrete data in the spatial domain, a graphical representation is proposed. The properties of graphs are studied using the eigenvalues and eigenvectors of the Laplacian matrix of the graph, extending DL techniques to the domain of graphs. The graph can be defined as,


(2) G=(V,E,A)

where *V* is the set of nodes, *E* is the set of edges, and *A* is the adjacency matrix of the graph.

Let *v*_*i*_ ∈ *V* denote a node and *e*_*ij*∉*E*_ denote an edge from *v*_*i*_ to *v*_*j*_. The neighborhood of node *v* is defined as,


(3) N⁢(v)={u∈V|(v,u)∈E}

The adjacency matrix *A* is the diagonal matrix *n*×*n*. The Laplacian matrix of a graph is defined as,


(4) L=D-A

where *L* is the Laplacian matrix and *D* is the degree matrix of the graph.

Since *L* is a symmetric matrix, it can be singular value decomposed (SVD) (Spielman, 2007), as follow,


(5) $$L=U\,\wedge \,U^{T}$$

where *U* = [*u*_0_,⋯,*u*_*N*−1_] ∈ *R*_*N*×*N*_ is the eigenvector matrix, ∧ = *diag*([λ_0_,⋯,λ_*N*_]) is the diagonal matrix.

GCN can be divided into two types of convolution including spectral convolution and spatial domain convolution. Spectral convolution is to filter both the convolutional network and graphical signals into the Fourier domain and then process them. Spatial domain convolution is to connect the nodes of the graph in the spatial domain, implement a hierarchy, and then perform convolution.

The spectral convolution of the graph signal is defined as,


(6) $$g_{\theta }*x=U\,g_{\theta }\,U^{T}\,x$$

where x ∈ *R^N^*, the filter is defined as *g*_θ_ = *diag*(θ), θ ∈ *R^N^* is parameter in the Fourier domain. *U* is consist of the eigenvectors from the normalized Laplacian matrix, *U* is defined as,


(7) $$L=I_{N}-D^{-\frac{1}{2}}\,A\,D^{-\frac{1}{2}}=U\,\wedge \,U^{T}$$

where ∧ is a diagonal matrix, which is consist of the eigenvalues of the Laplace matrix, *U^T^* is the Fourier transform of the graph.

To locate the filter in space and reduce its computational complexity, the filter is approximated using a truncated expansion of a K-order Chebyshev polynomial (Defferrard et al., 2016). The Chebyshev polynomial is defined as,


(8) $${\text{T}}_{k}\,\left(x\right)=2\,x\,T_{K-1}\,\left(x\right)-T_{K-2}\,\left(x\right)$$

where *T*_0_(*x*) = 1, *T*_1_(*x*) = *x*. Then, the signal *x* is filtered by a k-domain filter *y*, which is defined as,


(9) $$y=g_{\theta }\,\left(L\right)*x=\sum_{k=0}^{k}\theta_{k}\,T_{k}\,\left(\tilde{L}\right)\,x$$

where $\tilde{L}=2\,L/\lambda_{m\,a\,x}-I_{N}\,\lambda_{m\,a\,x}$ represents the largest eigenvalue of *L*.

**FIGURE 4:**
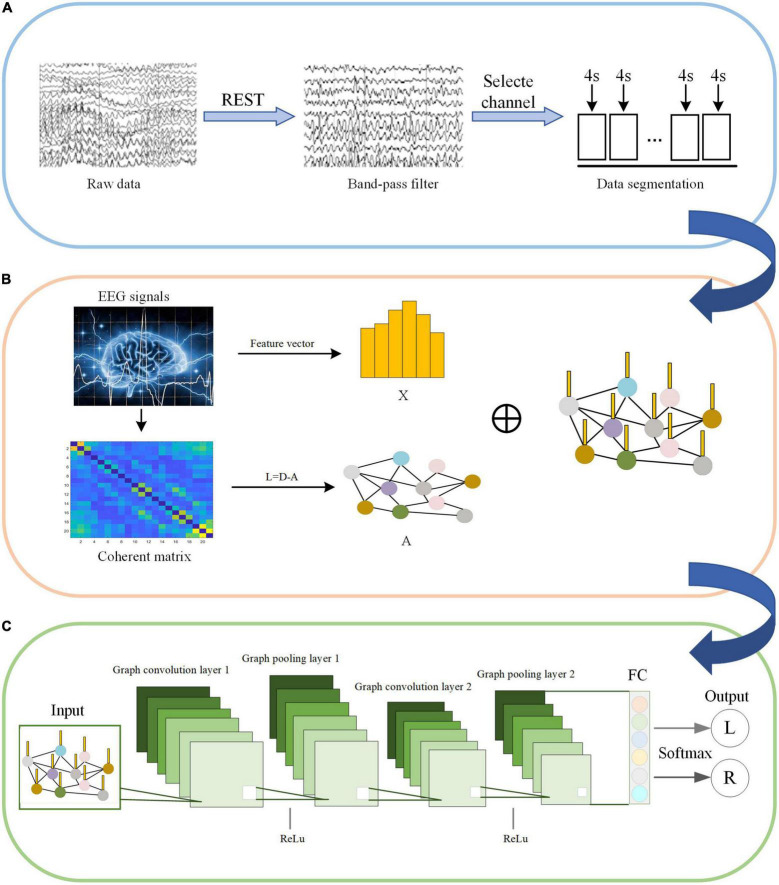
Motor imagery (MI) pattern recognition framework based on the coherence-based graph convolutional network (C-GCN) model. **(A)** Pre-processing of EEG. **(B)** Construction of graphical signals. **(C)** Specific GCN structure.

C-GCN is a model that combine coherence network with GCN. The framework consists of two main modules, including the construction of coherence-based graphical signals and pattern recognition for GCN. Before feeding into the C-GCN model, EEG data are first pre-processed as shown in Figure 4A. The input graphic signals of the C-GCN model integrate the temporal-frequency-spatial features from EEG data as shown in Figure 4B graphic signals of EEG data are implemented by formula (1) (9). After the graphic signals are constructed, EEG feature data is performed through two graph convolution layers, two Relu layers (Glorot et al., 2011), two graph pooling layers (Ouhmich et al., 2019), and one fully connected layer (Xu et al., 2022c) to complete the MI tasks classification as shown in Figure 4C.

The input of the C-GCN model is the pre-processed EEG time series. The X in Figure 4B represents the temporal-frequency features of EEG, the vertices in Figure 4B represent the EEG channels, and the edges connecting the vertices represent the coherence connectivity between electrodes. The purpose of performing graph convolution operations is to extract more discriminative EEG features. The graph convolutional layer is the core layer of C-GCN. To increase the non-linearity of the C-GCN model, the Relu function (Nair and Hinton, 2010) is used to mitigate the appearance of fitting problems. When the strength of the information is greater than a certain threshold, the valve is opened and the information is passed, otherwise, the valve is closed and the information is discarded. Graph pooling is a necessary module for GCN to perform classification, this module aggregates the previous results to obtain a smaller-scale representation of the graph. Graph pooling can be described as follows,


(10) $$P:\left(G=\left(V,E,A\right),Y\right)\to \left(G^{\prime }=\left(V^{\prime },E^{\prime },A^{\prime }\right),Y^{\prime }\right)$$

where the array of graph G and corresponding node feature matrix Y are mapped to a smaller array of graph G′ and corresponding node feature matrix Y′. In a multilayer GCN, the operation of the pooling layer is correspondingly expressed as,


(11) $$P:\left(G_{l}=\left(V_{l},E_{l},A_{l}\right),{\text{H}}^{\left(l\right)}\right)$$

$$\to \left(G_{l+1}=\left(V_{l+1},E_{l+1},A_{l+1}\right),{\text{H}}^{\left(l+1\right)}\right)$$

Then, a fully connected (FC) output layer is employed for integrating global information from the graphs of the previous localization filters. Finally, the Softmax function (Han et al., 2018) is used for classification and recognition. In the C-GCN model, the cross-entropy loss function is used to optimize the network parameters, and the cross-entropy loss is expressed as follows,


(12) $$L\,o\,s\,s=-\sum_{x}\left(p\,\left(x\right)\,\log q\,\left(x\right)+\left(1-p\,\left(x\right)\right)\,\log\left(1\,q\,\left(x\right)\right)\right)+\lambda \,K\,\left(w\right)$$

where *p*(*x*) denotes the true value of the training data, *q*(*x*) denotes the predicted value of the training data, *K*(*w*) is used to evaluate the model complexity and λ*K*(*w*) is aimed at preventing over fitting of the model. In summary, the EEG data based on the MI tasks are trained and tested in C-GCN to obtain classification recognition results. Algorithm 1 is a summary of the classification training steps of the C-GCN model.

**Algorithm 1:** Training procedure of the C-GCN model.

**Require:** the pre-processed EEG signals, the class labels corresponding to the EEG signals, the numbers of Chebyshev polynomial order k; **Ensure:** The desired model parameters of C-GCN; 1: **for** pre-processed EEG signals **do** 2: $C_{x\,y}=\frac{{\left|P_{x\,y}\,\left(f\right)\right|}^{2}}{P_{x\,x}\,\left(f\right)\,P_{y\,y}\,\left(f\right)}$ 3: **end for** 4: **for** graphical signals **do** 5: $L=I_{N}-D^{-\frac{1}{2}}\,A\,D^{-\frac{1}{2}}$ 6: $\tilde{L}=2\,L/\lambda_{m\,a\,x}-I_{N}\,\lambda_{m\,a\,x}$ 7: $T_{k}\left(\tilde{L}\right)\left(k=0,1,\cdots ,K-1\right)$ 8: $\sum_{K=0}^{K-1}\theta_{k}\,T_{k}\,\left(\tilde{L}\right)\,x$ 9: **end for** 10: Calculate the results of convolution after activation, FC layer, and Loss.

## 4. Results and discussions

### 4.1. C-GCN

**FIGURE 5:**
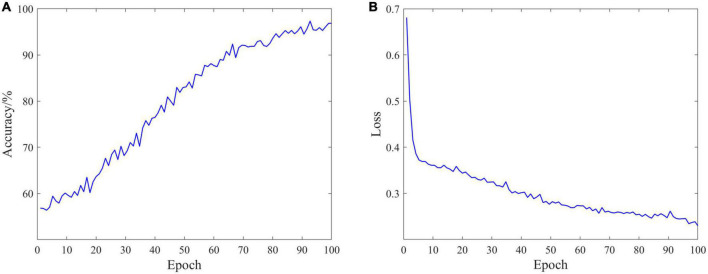
Classification performance of the coherence-based graph convolutional network (C-GCN) model. **(A)** Accuracy distribution of the C-GCN model. **(B)** Loss distribution of the C-GCN model.

**FIGURE 6:**
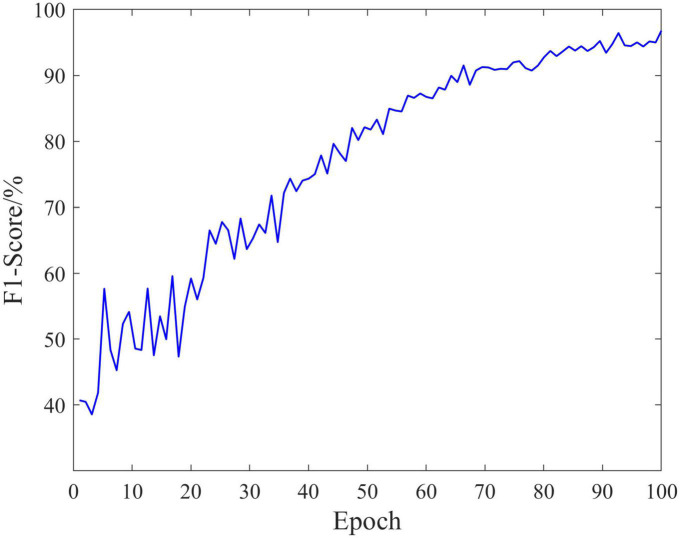
F1-Score of the coherence-based graph convolutional network (C-GCN) model.

The classification performance is an important measure of data quality, and can also provide ideas for the rehabilitation of SCI patients. In the experiment, EEG data of 18 SCI patients have been trained and validated on the proposed C-GCN model with the cross-validation method. The 90% of the data set have been employed for training and the 10% of the data set have been used for validation. The parameters of the model and the experimental results are shown in Table 1. In the C-GCN model, the accuracy of SCI patients can achieve 96.85%. The accuracy and Loss are shown in Figure 5, the F1-Score is shown in Figure 6. The experimental results show that the C-GCN model has a high signal-to-noise ratio, good adaptability, and robustness to individual specificity. The model can guide the rehabilitation training and subsequent treatment of SCI patients.

**TABLE 1:** Parameters values and experimental results of the coherence-based graph convolutional network (C-GCN) model.

Label	Parameters	Value
1	Num_epochs	100
2	Batch_size	512
3	Regularization	0.001
4	Dropout	0.50
5	Learning_rate	0.01
6	Accuracy	96.85%
7	Loss	0.23
8	F1-Score	96.78%

**FIGURE 7:**
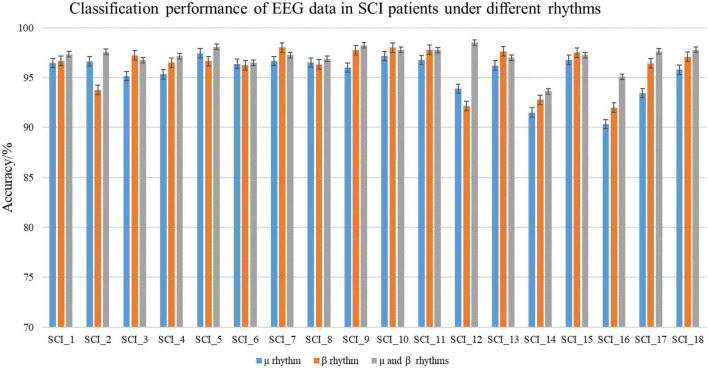
Classification performance of spinal cord injury electroencephalogram (SCI EEG) in the coherence-based graph convolutional network (C-GCN) model. This includes the classification accuracy under μ rhythm, β rhythm, μ and β rhythms.

To verify the difference of EEG data in MI tasks at different frequencies and the superiority of the C-GCN model, this paper also conducted experiments on μ rhythm, β rhythm, μ and β rhythms of 18 SCI patients respectively, the experimental results are shown in Table 2. In Figure 7, good classification accuracy can be obtained in μ rhythm, β rhythm, μ and β rhythms. There are slight differences in the classification results at different frequencies. In the vast majority of these cases, the classification accuracy of the MI tasks under μ and β rhythms is higher than that under μ or β rhythms alone, and the classification results of the MI tasks under β rhythms are higher than those under μ rhythms. This result indicates that the μ and β rhythms contains more information on MI in SCI patients, the MI information in the μ rhythm is less than that in the β rhythm. The information contained in the μ and β rhythms is more valuable for the rehabilitation research of SCI patients. The intra-individual classification accuracy of SCI patients is not significantly different from the overall classification accuracy. Table 2 indicates that the proposed C-GCN model has very strong adaptability and can mitigate the effects due to individual differences and the number of data samples.

**TABLE 2:** Classification accuracy of spinal cord injury (SCI) patients in the coherence-based graph convolutional network (C-GCN) model at different rhythms.

Subjects	Method	Accuracy%		
		**μ rhythm**	**β rhythm**	**μ and β rhythms**
SCI_1	C-GCN	96.45	96.68	97.33
SCI_2	C-GCN	96.63	93.75	97.58
SCI_3	C-GCN	95.15	97.23	96.75
SCI_4	C-GCN	95.33	96.50	97.15
SCI_5	C-GCN	97.43	96.65	98.08
SCI_6	C-GCN	96.38	96.25	96.50
SCI_7	C-GCN	96.65	98.00	97.25
SCI_8	C-GCN	96.50	96.33	96.88
SCI_9	C-GCN	96.00	97.75	98.23
SCI_10	C-GCN	97.15	98.00	97.78
SCI_11	C-GCN	96.75	97.78	97.75
SCI_12	C-GCN	93.88	92.15	98.50
SCI_13	C-GCN	96.23	97.63	97.00
SCI_14	C-GCN	91.50	92.78	93.63
SCI_15	C-GCN	96.78	97.50	97.25
SCI_16	C-GCN	90.33	92.00	95.08
SCI_17	C-GCN	93.45	96.43	97.63
SCI_18	C-GCN	95.78	97.08	97.78
Average	C-GCN	95.47	96.14	97.12
Standard deviation	C-GCN	1.91	1.96	1.13

**FIGURE 8:**
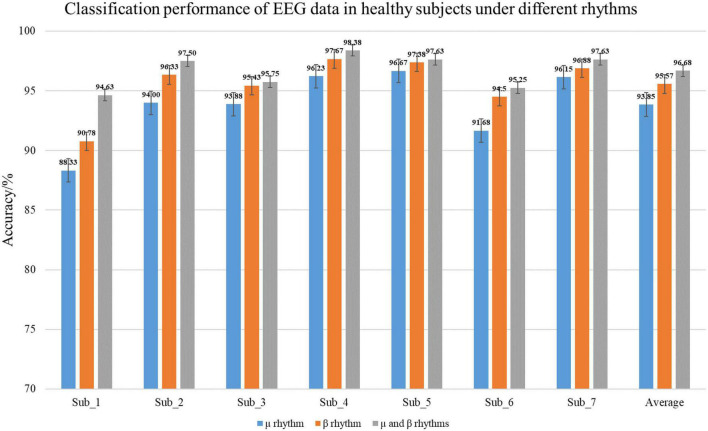
Classification performance of healthy subjects electroencephalogram (EEG) in the coherence-based graph convolutional network (C-GCN) model. This includes the classification accuracies under μ rhythm, β rhythm, μ and β rhythms.

In the experiment, healthy subjects as the control group. The classification accuracy of the EEG data under different rhythms in the C-GCN model for healthy subjects in Table 3. To observe the experimental results of the healthy subjects more visually, the classification results are presented in the form of histograms. In Figure 8, the experimental results of healthy subjects at different rhythms have the same regularity as those of SCI patients. The highest classification accuracy is obtained at the μ and β rhythms, followed by the second highest classification accuracy at the β rhythm and the lowest at the μ rhythm. Analysis of the mean accuracies revealed that the classification accuracy of SCI patients is slightly higher than that of healthy subjects at either rhythm and that the difference in accuracy between rhythms is lower in SCI patients than in healthy subjects. Combined with the self-assessment form of the subjects’ EEG acquisition procedure and the SCI pathology analysis, it is found that SCI is more focused during the EEG acquisition experiment and the quality of the collected EEG data is higher. Whereas healthy subjects have more active minds and are more easily influenced by their surroundings.

**TABLE 3:** Classification accuracy of healthy subjects in the coherence-based graph convolutional network (C-GCN) model at different rhythms.

Subjects	Method	Accuracy%		
		**μ rhythm**	**β rhythm**	**μ and β rhythms**
Sub_1	C-GCN	88.33	90.78	94.63
Sub_2	C-GCN	94.00	96.33	97.50
Sub_3	C-GCN	93.88	95.43	95.75
Sub_4	C-GCN	96.23	97.67	98.38
Sub_5	C-GCN	96.67	97.38	97.63
Sub_6	C-GCN	91.68	94.50	95.25
Sub_7	C-GCN	96.15	96.88	97.63
Average	C-GCN	93.85	95.57	96.68
Standard deviation	C-GCN	2.78	2.21	1.34

**FIGURE 9:**
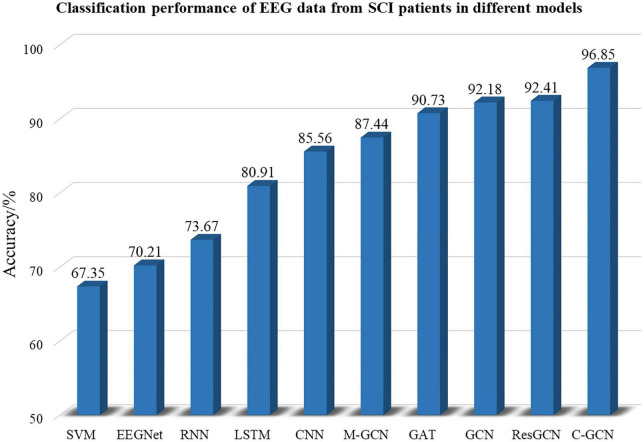
Classification performance of spinal cord injury electroencephalogram (SCI EEG) in different classification models. These include SVM, EEGNet, RNN, LSTM, CNN, GCN, M-GCN, GAT, ResGCN, and C-GCN models.

To verify the high performance of the model, this paper compares SVM (Kaper et al., 2004), EEGNet (Lawhern et al., 2018), RNN (Patnaik et al., 2017), LSTM (Wang et al., 2018), CNN (Zhou et al., 2018), M-GCN (Xu et al., 2022b), GAT (Demir et al., 2022), traditional GCN, and ResGCN with C-GCN. 18 SCI patients’ EEG data are involved in the training and testing of the above models. SVM is a traditional machine learning method whose classification performance may be affected by the extracted features and configuration parameters. EEGNet uses deep separable convolution to build EEG-specific models. RNNs memorize the previous information and apply it to the computation of the current output. LSTM is a special type of RNN that learns long-term dependent information. CNN can improve performance, but cannot effectively use the spatial information of EEG data. Traditional GCN only considers the spatial location relationships of channels, without considering the intrinsic connections of brain functions. M-GCN and ResGCN are improve patterns based on the traditional GCN. GAT, which helps to focus on the important information in the data, is a combination of graph neural network and attention layer. By selecting the optimal model parameters, the classification performance of each model can achieve the highest level. The classification results of SCI patient EEG data for the MI tasks in the SVM, EEGNet, RNN, LSTM, CNN, GCN, and C-GCN models are shown in Figure 9. The highest classification accuracy of 96.85% can been obtained from the C-GCN model. Compare with other models, the C-GCN model is 29.50% higher than the lowest SVM model.

### 4.2. Coherence networks

**FIGURE 10:**
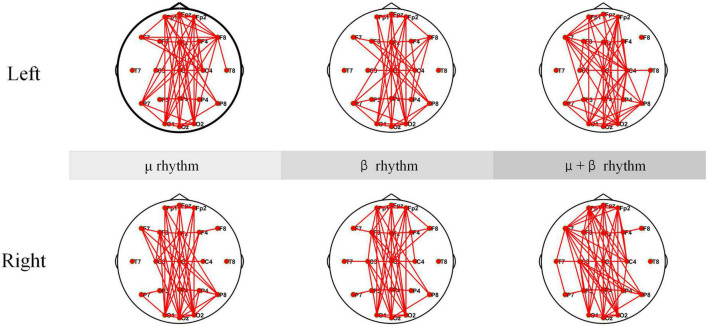
Coherence connectivity maps of motor imagery (MI) tasks in different frequency bands for spinal cord injury electroencephalogram (SCI EEG). These include left-hand and right-hand coherence connections under μ rhythm, β rhythm, μ and β rhythms.

Electroencephalogram coherence can generate network and functional integration information across brain regions. Any pair of EEG signals may be coherent in some frequency bands and incoherent in others. In the experiment, EEG coherence under μ rhythm, β rhythm, μ and β rhythms of SCI patients are analyzed separately, and the results are shown in Figure 10. According to the coherence network connectivity maps of SCI patients, it is found that SCI patients appear more obviously lateralized as well as long-range connections in the frontal-occipital lobe when performing left-hand and right-hand MI tasks. During performing the left-hand MI task, the connection between the frontal lobe (F8) and the parietal lobe (C4) is stronger under μ rhythm. The connection between F8 and C4 is weakened under β rhythm. In the μ and β rhythms, in addition to a strong connection at C4, the connection between the left frontal lobe (F7) and the right brain region becomes stronger. For right-handed subjects, the left brain also participates in processing relevant information during the execution of left-hand MI tasks to ensure task completion. When performing right-hand MI, the connectivity between the F3 and the C3 is enhanced under μ and β rhythms. In μ and β rhythms, the F3 and the C3 have a stronger connection. Meanwhile, the connectivity between the F7 and the parietal and occipital lobes is enhanced. In summary, C4 and its nearby electrode connectivity are enhanced when performing left-hand MI. C3 and its nearby electrode connectivity are enhanced when performing right-hand MI.

**FIGURE 11:**
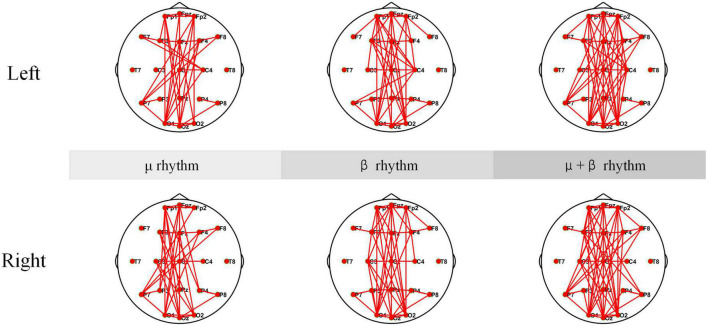
The Coherence connectivity maps of motor imagery (MI) tasks in different frequency bands for healthy subjects electroencephalogram (EEG). These include left-hand and right-hand coherence connections under μ rhythm, β rhythm, μ and β rhythms.

The coherence networks of healthy subject’s EEG data under μ rhythm, β rhythm, μ and β rhythms have shown in Figure 11. Healthy subjects have shown more significant laterality when performing the MI tasks. When performing left-hand MI, the connectivity is stronger in the parietal lobe (C4), and some electrodes in the left brain (e.g., F7) are also stronger connected to the right brain. In particular, C4 connectivity is strongest within the μ and β rhythms, followed by the β rhythm, and the μ rhythm is weakest in comparison. When performing right-hand MI, the connectivity of the parietal lobe (C3) and P7 is enhanced in the μ rhythm. The C3 is enhanced and the P7 connection is weakened under β rhythm. The connectivity of the C3 connection is strongest and the P7 connection is also enhanced under μ and β rhythms. In summary, C4 and partial electrode connectivity in the left brain is enhanced during the left-hand MI. C3 connectivity is enhanced during the performance of right-hand MI.

The coherence network in Figures 10, 11 have shown that the connection density of each electrode in SCI patients is significantly higher than healthy subjects. The connections are mainly existed on the prefrontal and occipital lobes. The Fp1, Fpz, and Fp2 have stronger connectivity than the other electrodes under μ rhythm, β rhythm, μ and β rhythms. The F7 in the SCI patients show higher connectivity density than in the healthy subjects under μ and β rhythms. Between the prefrontal and occipital lobes, the SCI patients have significantly more long-range connections than the healthy subjects. It can be inferred that the motor functional areas and sensory functional areas are damaged after SCI. The long-range connections existing between the frontal and occipital lobes of SCI patients are blocked. The cortical functional reorganization, neural activity increases, and functional compensation occurs in related brain areas. The differences of the coherence network between SCI patients and healthy subjects can be used to evaluate SCI, it is important for the clinical rehabilitation of SCI.

## 5. Conclusion

Spinal cord injury brings a lot of inconvenience to patients’ life. It is necessary to provide effective and scientific rehabilitation treatment methods. MI-based BCI system plays an increasingly important role in the rehabilitation treatment of SCI patients. The C-GCN model has been proposed to be applied for MI-based BCI system, which mainly consists of two parts, coherence network and GCN. The coherence network can analyze the intrinsic functional connectivity of the brain and fully exploit the relevant information between channels. GCN can connect the graphical information based on the functional connectivity of the brain to the fully connected layer and can learn the information of the surrounding nodes in the graphical signals. The C-GCN method combines the coherence network with GCN, retains the advantages of the two networks, and provides a guarantee for the classification and recognition of MI tasks in SCI patients. Specifically, the proposed algorithm uses a coherence matrix to characterize the relationship between channels, EEG features as graphical data and finally performs MI tasks classification recognition. The experiments are conducted in SCI patients and healthy subjects, the highest classification accuracy for the MI tasks in SCI patients is 96.85%, and the results are better than with six other classifiers. The average individual accuracy under μ rhythm for the MI pattern recognition in SCI patients is 95.47%, the average individual accuracy under β rhythm is 96.14%, and the average individual accuracy under μ and β rhythms is 97.12%. These experiments have proved that the C-GCN approach is reliable and effective. Furthermore, the C-GCN approach can provide a new strategy for the rehabilitation of SCI patients.

## Data availability statement

The datasets presented in this article are not readily available because the article data involves ethics and cannot be disclosed. Requests to access the datasets should be directed to FX, xfz@qlu.edu.cn.

## Ethics statement

The study protocols have been approved by the Medical Ethics Committee of Qilu Hospital, Cheeloo College of Medicine, Shandong University [No. KYLL-2020(KS)-475] on the Mar 25, 2020. The patients/participants provided their written informed consent to participate in this study.

## Author contributions

FX and HL have contributed to the conception and design of the study. HL, ML, XY, YZ, JZ, CW, and XC have collected the data, processed the data, and performed the experiments. HL has drafted the manuscript. FX, CF, YZ, and JL have reviewed the manuscript. All authors contributed to the article and approved the submitted version.
